# Synchronous double primary hepatocellular carcinoma and intrahepatic cholangiocarcinoma in a single patient with chronic hepatitis B: two case reports and literature review

**DOI:** 10.3389/fonc.2025.1507454

**Published:** 2025-06-16

**Authors:** Pengcheng Wei, Nan Kang, Chen Lo, Yongjing Luo, Jie Gao, Jiye Zhu, Zhao Li

**Affiliations:** ^1^ Department of Hepatobiliary Surgery, Peking University People’s Hospital, Beijing, China; ^2^ Beijing Key Surgical Basic Research Laboratory of Liver Cirrhosis and Liver Cancer, Peking University People’s Hospital, Beijing, China; ^3^ Peking University Center of Liver Cancer Diagnosis and Treatment, Peking University People’s Hospital, Beijing, China; ^4^ Department of Pathology, Peking University People’s Hospital, Beijing, China; ^5^ Peking University Institute of Organ Transplantation, Peking University People’s Hospital, Beijing, China

**Keywords:** double primary hepatic cancer, hepatocellular carcinoma, intrahepatic cholangiocarcinoma, chronic liver disease, hepatitis B virus

## Abstract

Simultaneous occurrence of primary hepatocellular carcinoma (HCC) and intrahepatic cholangiocarcinoma (ICC) is rare. We report two cases of synchronous double primary HCC and ICC (sdpHCC-ICC), both associated with chronic hepatitis B. Case 1 involves a 63-year-old man whose liver lesions were incidentally found during routine screening. Preoperative imaging revealed lesions in the S4 and S5 liver segments, with postoperative confirmation of sdpHCC-ICC. He received hepatic arterial infusion chemotherapy (HAIC) and transcatheter arterial chemoembolization (TACE) combined with gemcitabine and oral S-1 over 26 months, with no recurrence observed. Case 2 describes a 48-year-old woman presenting with right upper abdominal pain. Preoperative imaging identified a lesion at the S6/7 and S8 junction, later confirmed as sdpHCC-ICC. Postoperative TACE was performed at 1.5 and 3 months, and lenvatinib was introduced at 3.5 months. She remained recurrence-free at the 21-month follow-up. While the precise pathogenesis of sdpHCC-ICC remains unclear, chronic HBV infection plays a pivotal role. Surgical resection remains the primary treatment, though prognosis is generally poor due to the ICC component.

## 1 Introduction

Hepatocellular carcinoma (HCC) and intrahepatic cholangiocarcinoma (ICC) are the two most common pathological types of primary liver cancer, with HCC accounting for 75% to 85% and ICC for 10% to 15% (1, 2). Mixed hepatocellular carcinoma, containing both HCC and ICC components, is rare. In 1949, Allen et al. classified this tumor into three subtypes (3): Type A, where HCC and ICC grow independently in different liver regions; Type B, where both components form a continuous tumor; and Type C, where both components mix within the same tumor. In 1985, Goodman et al. proposed another classification (4): Type I for collision tumors, Type II for transitional tumors, and Type III for fibrolamellar tumors. Allen Type A and Goodman Type I are known as synchronous double primary hepatocellular carcinoma and intrahepatic cholangiocarcinoma (sdpHCC-ICC), with an incidence of less than 0.8% in primary liver cancer (5–7).

Surgical resection is the preferred treatment for sdpHCC-ICC. However, due to challenges in preoperative diagnosis, some patients are diagnosed at an advanced stage or are undergoing other localized treatments, resulting in missed surgical opportunities and poor prognosis (8). Reports on sdpHCC-ICC are scarce, but studies indicate that hepatitis B virus (HBV) and hepatitis C virus (HCV) infections are associated with its development (9–11). In this study, we reviewed the literature and analyzed two cases of sdpHCC-ICC associated with chronic HBV infection from our center, aiming to enhance clinical understanding and provide insights into its diagnosis and management. In addition to comprehensive clinical data—including preoperative imaging, histopathological features, and immunohistochemical profiles—we incorporated next-generation sequencing (NGS) to characterize molecular alterations and explore potential pathogenic mechanisms and therapeutic targets. Compared to prior case reports primarily focused on pathological findings, this study integrates clinical, pathological, and molecular perspectives to enhance understanding of the biological heterogeneity of sdpHCC-ICC and support the development of precise diagnostic and therapeutic strategies.

## 2 Case presentation

### 2.1 Case 1

A 63-year-old man was found to have hepatic lesions during an abdominal ultrasound examination performed as part of a routine physical check-up six months ago. Due to the small size of the lesions, regular follow-up was recommended. A recent review at an outside hospital revealed lesion enlargement, though the patient reported no symptoms such as abdominal pain, distension, nausea, vomiting, or significant weight change. He had a long history of chronic hepatitis B without antiviral treatment and began oral entecavir after admission. He also had a history of alcohol consumption but no history of metabolic diseases. Laboratory tests showed a normal platelet count and liver function indicators. The results also included positive HBV surface antigen (HBsAg), positive anti-HBV core antibody (anti-HBc), an HBV-DNA level of 928 IU/ml, and a negative anti-HCV antibody. Tumor marker results were as follows: alpha-fetoprotein (AFP) 2.91 ng/ml, protein induced by vitamin K antagonist-II (PIVKA-II) 45.08 mAU/ml, carcinoembryonic antigen (CEA) 1.61 ng/ml, and carbohydrate antigen 19-9 (CA19-9) 12.10 U/ml.

**Figure 1:**
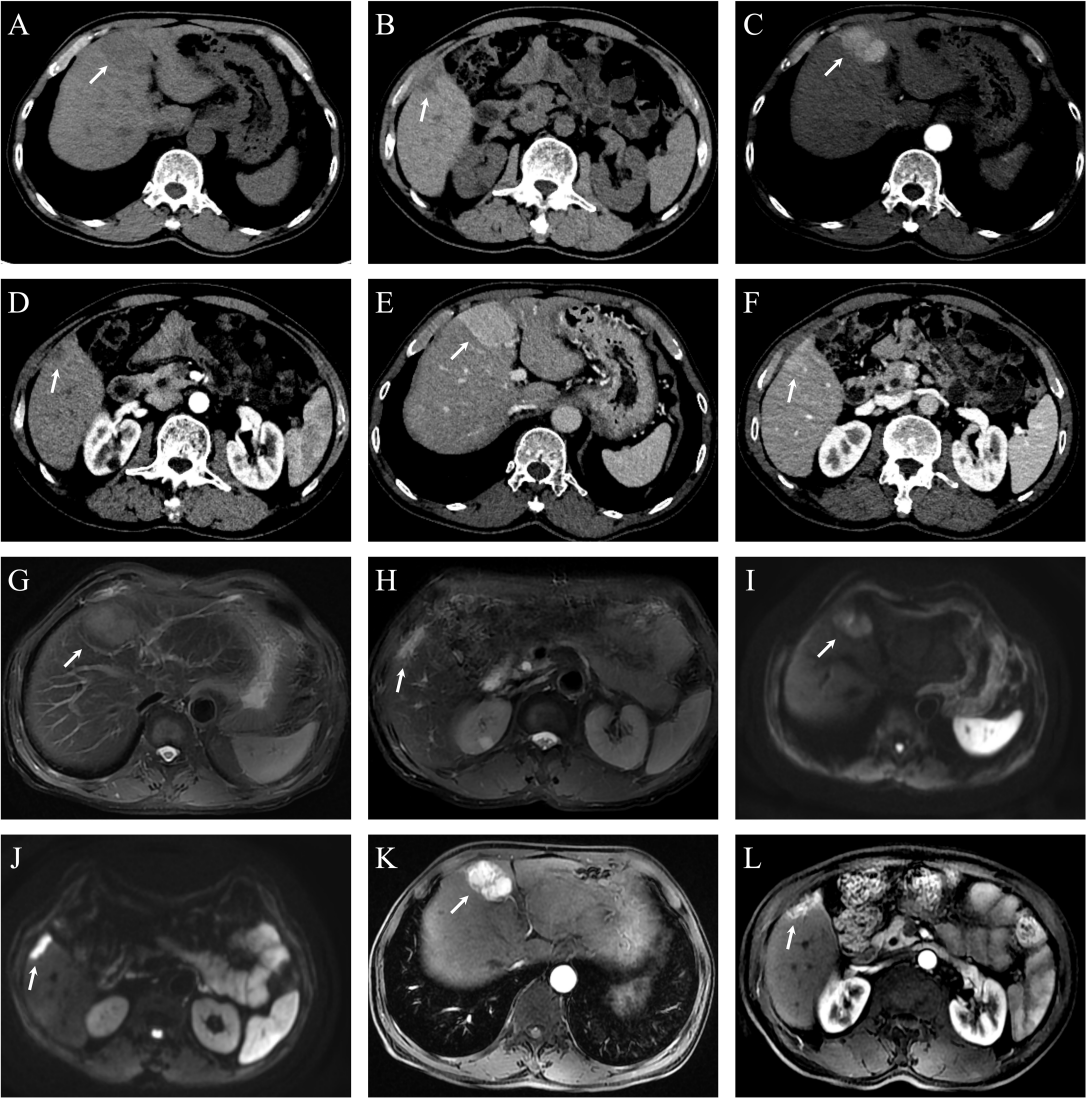
Dynamic contrast-enhanced CT (DCE-CT) and dynamic contrast-enhanced MRI (DCE-MRI) of hepatic lesions in Case 1. **(A)** Non-contrast CT shows a hypodense lesion in segment 4 (S4), measuring approximately 5.1 × 3.7 cm. **(B)** A smaller hypodense lesion is observed in segment 5 (S5), with a diameter of approximately 0.6 cm. **(C)** In the arterial phase, the S4 lesion demonstrates heterogeneous enhancement. **(D)** The S5 lesion shows subtle peripheral nodular enhancement in the arterial phase, though its margin is not well-defined. **(E)** The S4 lesion exhibits homogeneous and persistent hyperenhancement in the portal venous phase, with enhancement intensity exceeding that of the surrounding liver parenchyma. **(F)** The S5 lesion appears slightly hypodense in the portal venous phase. **(G)** Axial T2-weighted fat-suppressed MRI (RTr Ax T2 FS PROP) reveals an ill-defined, mildly hyperintense lesion in S4. **(H)** In the same sequence, a subcapsular, elongated hyperintense signal is noted in S5. **(I)** Diffusion-weighted imaging (RT AX DWI) demonstrates mildly increased signal intensity in the S4 lesion. **(J)** The S5 lesion shows markedly increased signal intensity on DWI, indicating restricted diffusion. **(K)** Post-contrast axial T1-weighted LAVA-Flex sequence shows marked heterogeneous enhancement of the S4 lesion with visible feeding vessels and a central non-enhancing area suggestive of necrosis. **(L)** The S5 lesion shows strong homogeneous enhancement in the same sequence.

**Figure 2:**
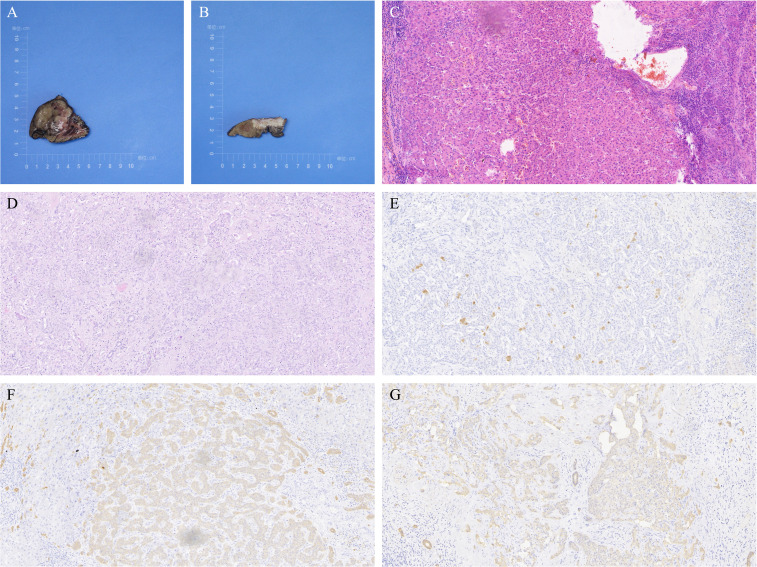
Liver tumor resection specimens from Case 1. **(A)** Resected specimen of the tumor in segment S4, identified as well-differentiated hepatocellular carcinoma (HCC), measuring 3.7×3.2×3.6 cm. **(B)** Resected specimen of the tumor in segment S5, identified as moderately differentiated intrahepatic cholangiocarcinoma (ICC), measuring 3.2×3.5×1.1 cm. **(C)** Hematoxylin and eosin (H&E) staining of HCC in segment S4 at 200x magnification. **(D)** H&E staining of ICC in segment S5 at 200x magnification. **(E)** Immunohistochemistry (IHC) showing negative Arg staining in ICC from segment S5 at 200x magnification. **(F)** IHC showing weakly positive CK7 staining in ICC from segment S5 at 200x magnification. **(G)** IHC showing weakly positive CK19 staining in ICC from segment S5 at 200x magnification.

The patient completed Dynamic Contrast-Enhanced Computed Tomography (DCE-CT) and Dynamic Contrast-Enhanced Magnetic Resonance Imaging (DCE-MRI). DCE-MRI revealed a blood-rich space-occupying lesion in the S4 segment of the liver, measuring approximately 5.1×3.7 cm. The lesion exhibited inhomogeneous enhancement in the arterial phase and homogeneous enhancement in the venous phase. A subcapsular lesion with a strip-like low signal was observed in segment 5 of the liver, with ill-defined margins. On contrast-enhanced imaging, the lesion demonstrated marked enhancement in all phases, accompanied by mild atrophy of the surrounding hepatic parenchyma and capsular retraction. See **Figure 1**. On the 9th day of admission, laparoscopic resection of the S4 segment of the liver, partial resection of the S5 segment, and cholecystectomy were performed. Postoperative pathology revealed that the mass in the S4 segment of the liver was highly differentiated HCC, measuring 3.7 cm × 3.2 cm × 3.6 cm. Immunohistochemical results were: CK7 (–), CK19 (–), hepatocyte (+), AFP (+), Arg (+), and GPC3 (+).The mass in the S5 segment of the liver was moderately differentiated ICC, measuring 3.2 cm × 3.5 cm × 1.1 cm. Immunohistochemistry results were: CK7 (weakly +), CK19 (weakly +), hepatocyte (–), AFP (–), Arg (–), and GPC3 (–).The cholecystectomy specimen showed chronic cholecystitis, with no carcinoma observed at the severed end. The pathological diagnosis was consistent with sdpHCC-ICC. See **Figure 2**.

Following surgical resection, the patient received eight sessions of intra-arterial therapy, comprising five sessions of hepatic arterial infusion chemotherapy (HAIC) and three of transcatheter arterial chemoembolization (TACE), administered at 1 to 1.5-month intervals. In each session, 100 mg of oxaliplatin was infused via microcatheter into the right hepatic artery, followed by diagnostic embolization using 1–2 mL of ultra-fluid lipiodol. Gemcitabine (1400 mg) was co-administered during each cycle, along with oral S-1 as part of the combination chemotherapy regimen. At 26 months postoperatively, follow-up imaging showed no evidence of tumor recurrence.

### 2.2 Case 2

A 48-year-old woman was admitted to the hospital with a 3-week history of right upper abdominal pain. An external abdominal ultrasound suggested cirrhosis with multiple intrahepatic nodules. At admission, the patient experienced loss of appetite without abdominal distension, nausea, or vomiting, and had recently lost 4 kg of body weight. She had a 15-year history of chronic hepatitis B and started taking tenofovir orally in the past 3 weeks. She had no history of alcohol consumption or metabolic diseases. Laboratory tests showed normal platelet count and liver function indexes, positive HBsAg, positive HBV e antigen (HBeAg), positive anti-HBc, an HBV-DNA level of 103,000 IU/ml, and a negative anti-HCV antibody. Tumor marker results were as follows: AFP 652.00 ng/ml, PIVKA-II 23.55 mAU/ml, CEA 0.44 ng/ml, and CA19-9 52.80 U/ml.

**Figure 3:**
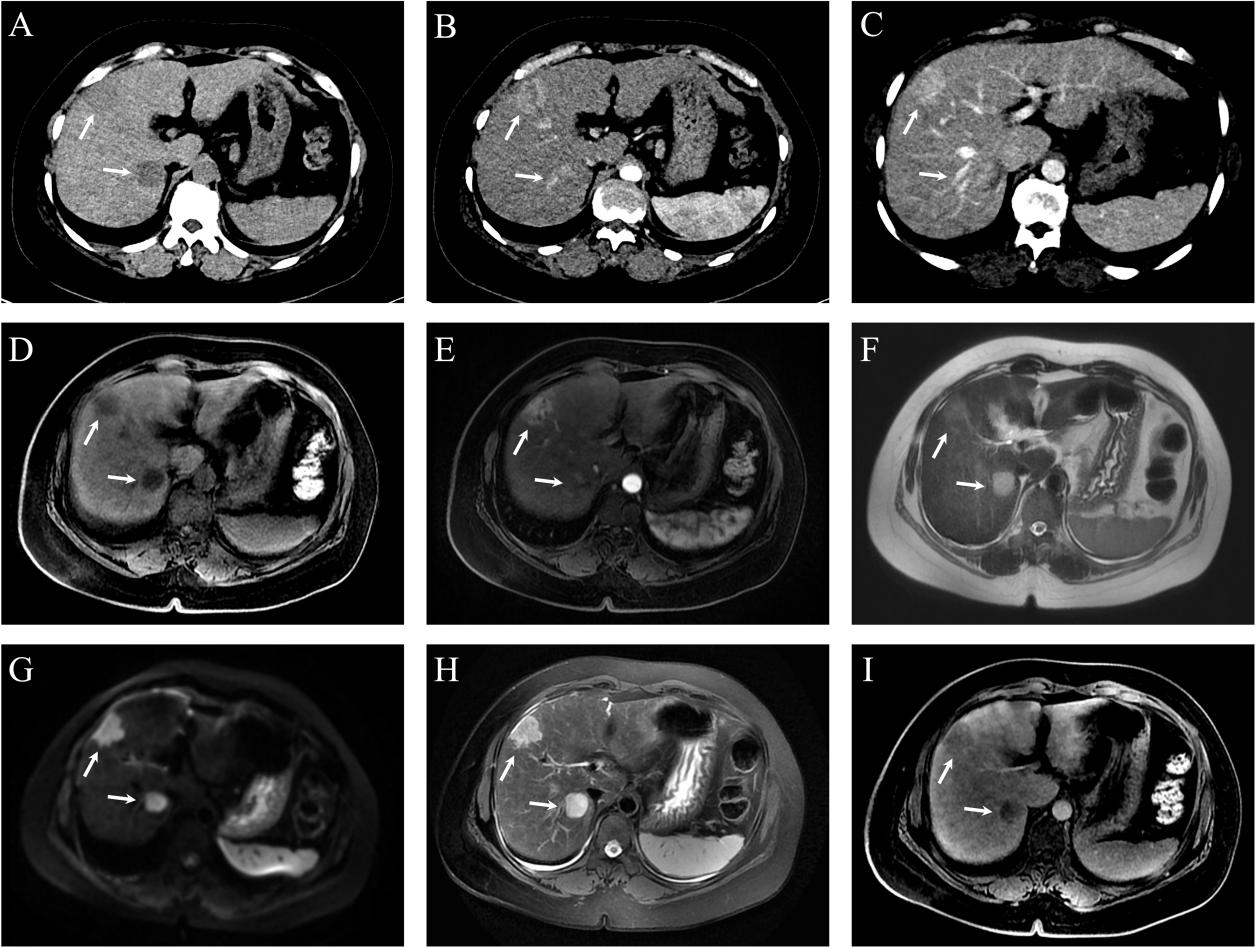
Dynamic contrast-enhanced CT (DCE-CT) and dynamic contrast-enhanced MRI (DCE-MRI) of hepatic lesions in Case 2. **(A)** Non-contrast CT shows two slightly hypodense, round-like lesions at the junction of segments 8 (S8) and 6/7 (S6/7), measuring approximately 3.5 × 2.5 cm (S8) and 3.1 × 2.4 cm (S6/7), respectively. **(B)** Both lesions demonstrate heterogeneous enhancement during the arterial phase. **(C)** Persistent enhancement is observed in the portal venous phase at the same locations. **(D)** Pre-contrast axial T1-weighted LAVA-Flex MRI reveals ill-defined, round-like lesions in S8 and at the S6/7 junction with mildly hyperintense T1 signal. **(E)** On contrast-enhanced LAVA-Flex MRI, the S8 lesion shows marked enhancement, while the S6/7 lesion demonstrates faint enhancement. **(F)** Axial SSFSE sequence shows a round hyperintense lesion at the S6/7 junction; the S8 lesion is less clearly visualized. **(G)** Diffusion-weighted imaging (DWI) reveals hyperintense signal in both S8 and S6/7 lesions, indicating restricted diffusion. **(H)** Fat-suppressed axial T2-weighted MRI (RTr Ax T2 FS PROP) shows clear, round, hyperintense lesions at the junction of segments 8 and 6/7. **(I)** On delayed-phase post-contrast LAVA-Flex MRI, both S8 and S6/7 lesions exhibit sustained enhancement, with intensity slightly higher than the surrounding liver parenchyma.

**Figure 4:**
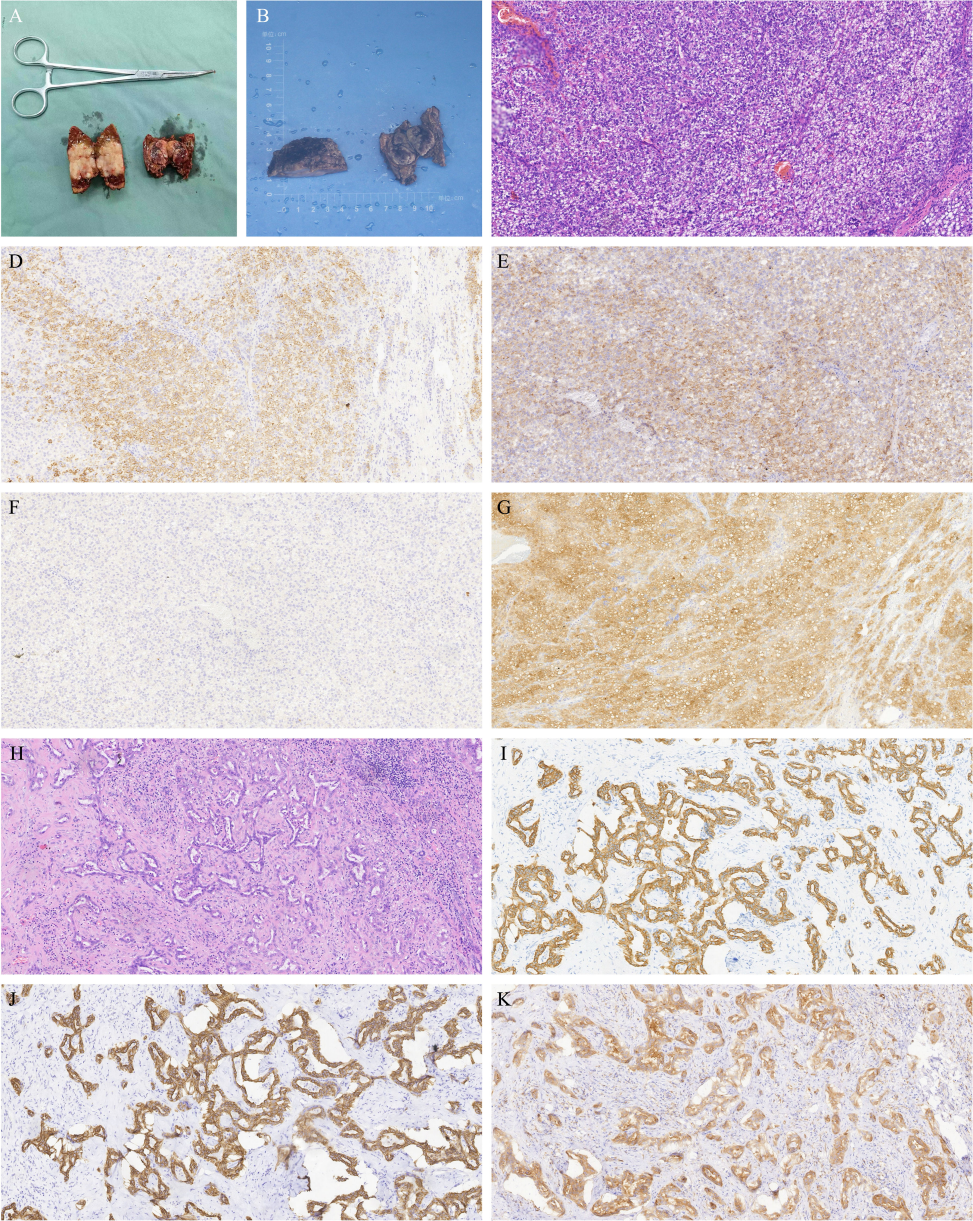
Liver tumor resection specimens from case 2. **(A, B)** The left specimen in both images is from segment S8, identified as moderately differentiated intrahepatic cholangiocarcinoma (ICC), measuring 3×2×2 cm. The right specimen is from segment S6/7, identified as moderately differentiated hepatocellular carcinoma (HCC), measuring 2.5×2×2 cm. **(C)** Hematoxylin and eosin (H&E) staining of HCC in segment S6/7 at 200x magnification. **(D)** Immunohistochemistry (IHC) showing positive hepatocyte staining in HCC from segment S6/7 at 200x magnification. **(E)** IHC showing positive GS staining in HCC from segment S6/7 at 200x magnification. **(F)** IHC showing focal positive GPC3 staining in HCC from segment S6/7 at 200x magnification. **(G)** IHC showing positive Arg staining in HCC from segment S6/7 at 200x magnification. **(H)** H&E staining of ICC in segment S8 at 200x magnification. **(I)** IHC showing positive CK7 staining in ICC from segment S8 at 200x magnification. **(J)** IHC showing positive CK19 staining in ICC from segment S8 at 200x magnification. **(K)** IHC showing positive GS staining in ICC from segment S8 at 200x magnification.

Both CT and MRI revealed a space-occupying lesion at the junction of hepatic segments S8 and S6/7, measuring approximately 3.5×2.5 cm. The lesion showed multiple vascular-like enhancements in the arterial phase and slightly higher enhancement in the venous phase compared to the surrounding hepatic parenchyma, initially suggesting HCC. Additionally, multiple regenerative nodules, the largest measuring about 0.7 cm, were present in the liver, some of which could not be ruled out as early-stage hepatocellular carcinoma. See **Figure 3**. On the 3rd day of admission, laparoscopic partial resection of liver segments S8 and S6/7 was performed. Postoperative pathology revealed that the mass in segment S8 was moderately differentiated ICC, measuring 3 cm × 2 cm × 2 cm, with immunohistochemical results: CK7 (+), CK19 (+), hepatocyte (–), GS (+), GPC3 (–), and Arg (–). The mass in segment S6/7 was moderately differentiated HCC, measuring 2.5 cm × 2 cm × 2 cm, with immunohistochemical results: CK7 (–), CK19 (–), hepatocyte (+), GS (+), GPC3 (+), and Arg (+). The pathological diagnosis was consistent with sdpHCC-ICC. See **Figure 4**.

The patient underwent two sessions of TACE at 1.5 and 3 months after resection, with epirubicin 30 mg as the chemotherapy drug. Starting at 3.5 months postoperatively, the patient began taking oral lenvatinib, which has been continued to date. There were no signs of recurrence at the 21-month postoperative follow-up.

## 3 Literature review

A review of 16 reported cases of sdpHCC-ICC, including two from the present study (**Table 1**), showed that patients were predominantly middle-aged to elderly (41–77 years), with a peak incidence between 50 and 70 years. The male-to-female ratio was approximately 1:1, suggesting no significant sex-related predisposition. HBV infection was the most common etiological factor (11/16), followed by HCV (3/16), with a minority lacking a history of viral infection. Underlying liver disease was primarily chronic hepatitis (8/16) or cirrhosis (5/16), supporting the hypothesis that chronic liver inflammation, especially of viral origin, contributes significantly to tumorigenesis. Imaging revealed that most HCC lesions were located in the right hepatic lobe (segments 5–8, ~75%), whereas ICC lesions were more diffusely distributed across segments 2–4 and junctional regions, suggesting that the two tumor components may originate from distinct biliary progenitor lineages. The average diameter of HCC lesions was generally larger than that of ICC (up to 10 cm vs. 5 cm), indicating that HCC often dominates the clinical manifestation. Symptomatically, 10 patients presented with liver mass or abdominal pain, whereas 6 were asymptomatic and diagnosed incidentally, emphasizing the importance of imaging-based detection and pathological confirmation, particularly immunohistochemistry, for accurate diagnosis.

**Table 1:** Reported cases of synchronous double primary hepatocellular carcinoma and intrahepatic cholangiocarcinoma (sdpHCC-ICC) undergoing surgical resection, including the present cases.

No.	Age	Gender	Viral infection	Underlying liver disease	Localization HCC/ICC	Size (cm) HCC/ICC	Symptom	Treatment	Follow-up	Prognosis	Author	Year
1	70	M	HCV	Chronic hepatitis	S4/S7	4/2.2	Liver mass	Segmentectomy	30 months	Alive without recurrence	Matsuda et al. (38)	2006
2	67	M	HCV	Liver cirrhosis	S7/S8	1.3/1.2	Liver mass	Partial resection + TACE	84 months	Recurrence occurred	Inaba et al. (24)	2007
3	66	F	HBV	Chronic hepatitis	S7/S2-3	N/A	Liver mass	Partial resection+ Chemotherapy	7 months	Alive without recurrence	Jung et al. (11)	2013
4	68	F	HBV	Chronic hepatitis	S5/S3	4.3/1.1	Liver mass	Segmentectomy	24 months	Alive without recurrence		
5	58	M	Negative	None	S5/S8	6/4	Abdominal pain	Partial resection	11 months	Alive without recurrence	Wu et al. (39)	2014
6	48	M	HBV	Chronic hepatitis	S5, S7/S2-3	5, 10/8	Liver mass	Segmentectomy + Chemotherapy	6 months	Alive without recurrence	Topaloğlu et al. (40)	2014
7	56	M	Negative	None	S7/S6	7/4.5	Right lumbago	Partial resection	12 months	Alive without recurrence	Hu et al. (10)	2016
8	41	M	HBV	Chronic hepatitis	S7/S6	4/1	None	Segmentectomy	8 months	Alive without recurrence	Zhou et al. (25)	2016
9	45	M	HBV	Liver cirrhosis	S6/S7	2.3/1	None	Segmentectomy	20 months	Alive without recurrence	Suzumura et al. (30)	2016
10	58	M	HBV, HCV	Chronic hepatitis	S7/S3	1.4/0.8	None	Partial resection	24 months	Alive without recurrence	Yamamoto et al. (28)	2018
11	49	F	HBV	Liver cirrhosis	S6/S4	2.5/5	Abdominal pain	Partial resection	16 months	Died of liver failure	Qu et al. (8)	2021
12	69	F	HBV	Liver cirrhosis	S4/S6	2.1/3	Abdominal pain	Partial resection	7 months	Recurrence occurred	Gao et al. (41)	2022
13	75	F	HCV	Liver cirrhosis	S8/S5	1.5/2	None	Segmentectomy	6 months	Alive without recurrence	Khessairi et al. (42)	2024
14	77	F	HBV	Chronic hepatitis	S5/S8, S4	1.5/3.6	None	Partial resection + Chemotherapy	12 months	Alive without recurrence	Fukuda et al. (43)	2024
15	63	M	HBV	Chronic hepatitis	S4/S5	3.7/3.5	None	Partial resection + Chemotherapy + HAIC + TACE	26 months	Alive without recurrence	Present Case 1	2024
16	48	F	HBV	Chronic hepatitis	S6-7/S8	2.5/3	Abdominal pain	Partial resection + TACE + Lenvatinib	21 months	Alive without recurrence	Present Case 2	2024

M, male; F, female; HCV, hepatitis C virus; HBV, hepatitis B virus; HCC, hepatocellular carcinoma; ICC, intrahepatic cholangiocarcinoma; N/A, not available; TACE, transarterial chemoembolization; HAIC, hepatic arterial infusion chemotherapy.

All patients underwent curative surgical resection, either via anatomical segmentectomy or partial hepatectomy. Six patients received postoperative adjuvant therapy, including TACE, HAIC, systemic chemotherapy, or targeted therapy. Within this subgroup, only one recurrence occurred during a follow-up period of 6 to 84 months, suggesting that adjuvant therapy may improve prognosis. In contrast, among the ten patients treated with surgery alone, two experienced recurrence and one died of liver failure, suggesting that postoperative adjuvant therapy may prolong disease-free survival (DFS) and overall survival (OS), despite the small sample size. The median follow-up duration was 15 months (range: 6–84 months), with most patients remaining recurrence-free within two years post-surgery. In the present study, both cases were HBV-related, with tumors located in segments S4/S5 and S6-7/S8, respectively. Both patients received surgical resection combined with adjuvant TACE plus HAIC and chemotherapy, or TACE plus lenvatinib, and remained recurrence-free at 21 and 26 months of follow-up, respectively. These findings further support the potential benefit of comprehensive treatment strategies in improving outcomes for HBV-related sdpHCC-ICC and offer clinical evidence to guide diagnostic and adjuvant treatment strategies in future practice.

## 4 Discussion

**Figure 5:**
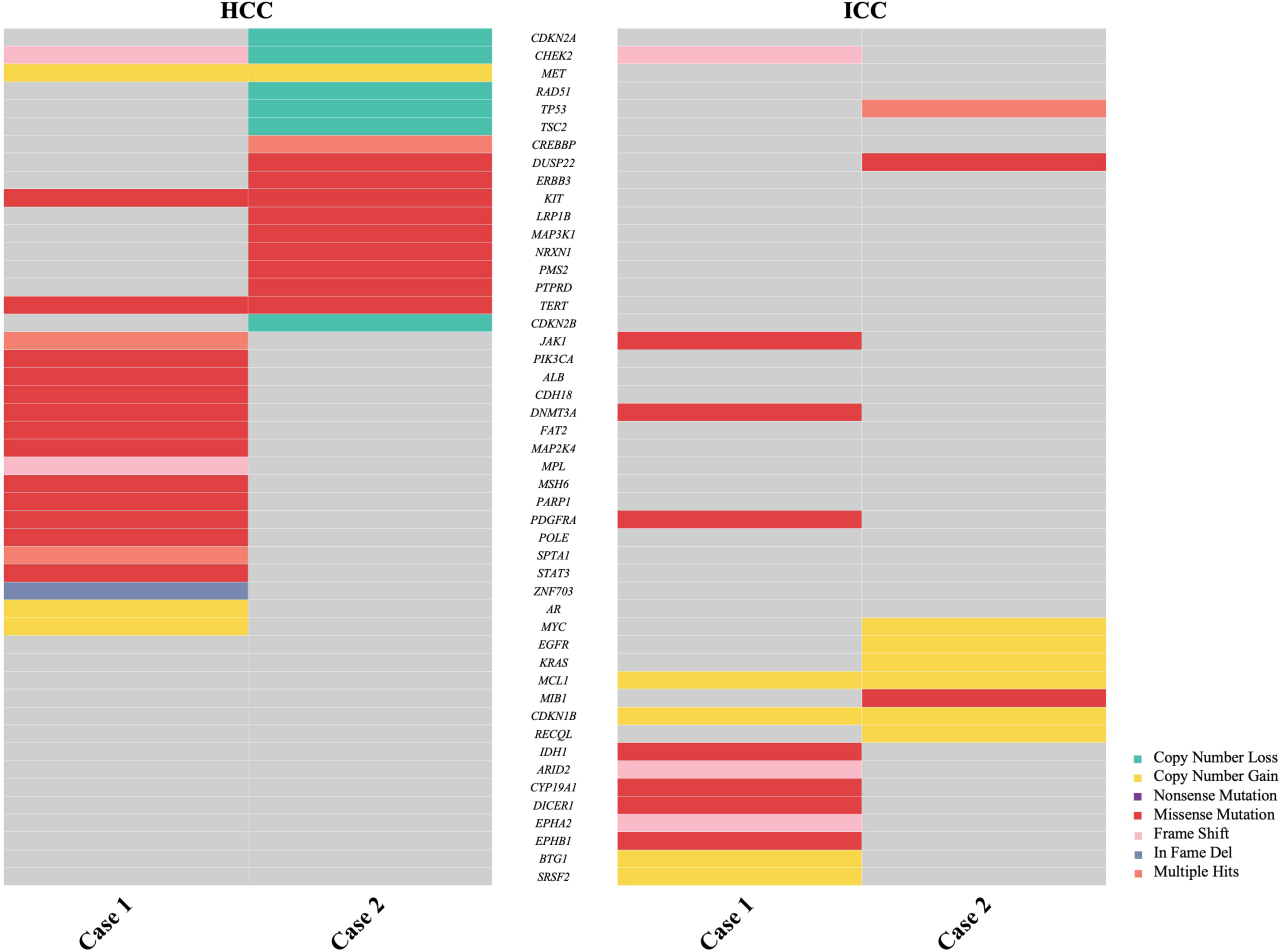
Comparison of next-generation sequencing (NGS) analysis of 1021 relevant genes in hepatocellular carcinoma (HCC) and intrahepatic cholangiocarcinoma (ICC) from two cases.

In 2004, the World Health Organization (WHO) excluded stand-alone HCC and ICC occurring simultaneously in the liver from combined hepatocellular-cholangiocarcinoma (cHCC-CCA). Since then, the classification mechanism for primary liver cancer has been continuously refined. The 2019 WHO Classification of Tumors of the Digestive System further clarified the classification of primary liver cancer based on molecular studies, identifying different pathogenic mechanisms for various pathological types (12, 13). The pathogenesis of sdpHCC-ICC, a rare primary liver cancer, is unclear, with long-standing controversy regarding the origin of tumor cells. One view suggests that liver tumor cells originate from hepatic progenitor cells (HPC) with bipotent differentiation potential, capable of differentiating into hepatocytes or cholangiocytes and undergoing malignant proliferation (14–16). Another view proposes that the tumor cells of HCC and ICC originate independently, or that HCC initially emerges and transforms into ICC, and vice versa (17). Xue et al. demonstrated that sdpHCC-ICC may have both monoclonal and polyclonal origins, with foci of different phenotypes originating from the same clone, suggesting a phenotypic shift (18). Genetic testing was conducted on the ICC and HCC lesions of the patients, with 1021 tumor-related genes sequenced using NGS. The details are provided in **Tables 2**, **3**, and **Figure 5**. In Case 1, the mutation profiles of the two tumor lesions shared only a PDGFRA and DNMT3A missense mutation and a CHEK2 frameshift mutation. In Case 2, the two tumor lesions shared only a DUSP22 missense mutation and a TP53 copy number loss in their mutation profiles. These findings indicate that the ICC and HCC lesions in these two cases likely arose from distinct clones.

**Table 2:** The results of the next-generation sequencing (NGS) analysis of 1021 relevant genes from the liver tumor resection specimen in Case 1.

Cancer Type	Gene	Mutation Result	Variant Type	Abundance	Mutation Grade
**ICC**	*DICER1*	p.F1677L	Missense mutation	19.8%	III
	*EPHB1*	p.P420S	Missense mutation	19.3%	III
	*ARID2*	p.R1754Efs*11	Frame shift	17.7%	III
	*CYP19A1*	p.M356V	Missense mutation	16.2%	III
	*IDH1*	p.R132C	Missense mutation	14.5%	I
	*EPHA2*	p.I619Mfs*13	Frame shift	12.3%	III
	*CDKN1B*	Amplification	Copy number gain	5.4%	III
	*MCL1*	Amplification	Copy number gain	5.2%	II
	*SRSF2*	Amplification	Copy number gain	4.2%	III
	*BTG1*	Amplification	Copy number gain	4.0%	III
	*PDGFRA*	p.A146V	Missense mutation	2.4%	III
	*DNMT3A*	p.R771Q	Missense mutation	2.2%	III
	*CHEK2*	p.R523Vfs*43	Frame shift	1.6%	II
	*JAK1*	p.R108Q	Missense mutation	1.0%	III
**HCC**	*ZNF703*	p.R222_S225del	In frame del	31.5%	III
	*TERT*	c.-58-u66C>T	Missense mutation	26.2%	III
	*STAT3*	p.Q361P	Missense mutation	21.5%	III
	*KIT*	p.Y221C	Missense mutation	21.4%	III
	*CDH18*	p.R689K	Missense mutation	20.0%	III
	*JAK1*	p.S703I	Missense mutation	14.1%	II
	*POLE*	p.R2016K	Missense mutation	3.9%	III
	*FAT2*	p.Q3494R	Missense mutation	3.8%	III
	*PIK3CA*	p.H1047R	Missense mutation	3.5%	II
	*MYC*	Amplification	Copy number gain	3.0%	III
	*MET*	Amplification	Copy number gain	2.8%	II
	*MAP2K4*	p.K357T	Missense mutation	2.5%	III
	*JAK1*	p.K924M	Missense mutation	2.3%	III
	*MSH6*	p.C779*	Missense mutation	2.2%	III
	*ALB*	p.E525Vfs*2	Missense mutation	2.1%	III
	*DNMT3A*	p.R771Q	Missense mutation	1.7%	III
	*AR*	Amplification	Copy number gain	1.7%	III
	*MPL*	p.P227Lfs*4	Frame shift	1.6%	III
	*PARP1*	p.A884T	Missense mutation	1.5%	III
	*PDGFRA*	p.A146V	Missense mutation	1.5%	III
	*SPTA1*	p.T635N	Missense mutation	1.5%	III
	*SPTA1*	p.D318G	Missense mutation	1.2%	III
	*CHEK2*	p.R523Vfs*43	Frame shift	1.1%	II

ICC, intrahepatic cholangiocarcinoma; HCC, hepatocellular carcinoma.

**Table 3:** The results of the next-generation sequencing (NGS) analysis of 1021 relevant genes from the liver tumor resection specimen in case 2.

Cancer Type	Gene	Mutation Result	Variant Type	Abundance	Mutation Grade
**ICC**	*DUSP22*	p.V129M	Missense mutation	32.5%	III
	*MIB1*	p.S300T	Missense mutation	19.5%	III
	*EGFR*	Amplification	Copy number gain	10.6%	II
	*TP53*	p.C176F	Missense mutation	8.9%	II
	*MYC*	Amplification	Copy number gain	7.8%	III
	*MCL1*	Amplification	Copy number gain	4.8%	II
	*RECQL*	Amplification	Copy number gain	4.6%	III
	*CDKN1B*	Amplification	Copy number gain	4.0%	III
	*KRAS*	Amplification	Copy number gain	3.0%	II
	*TP53*	Deletion	Copy number loss	1.4%	II
**HCC**	*PTPRD*	p.M1164I	Missense mutation	73.5%	III
	*KIT*	p.A777V	Missense mutation	41.3%	III
	*TERT*	c.-58-u66C>T	Missense mutation	32.6%	III
	*DUSP22*	p.V129M	Missense mutation	32.0%	III
	*CREBBP*	p.0?	Nonsense mutation	21.2%	III
	*NRXN1*	p.W183R	Missense mutation	19.9%	III
	*ERBB3*	p.Q1301K	Missense mutation	19.2%	III
	*LRP1B*	p.L700I	Missense mutation	17.8%	III
	*PMS2*	p.Q342R	Missense mutation	14.0%	III
	*MET*	Amplification	Copy number gain	2.8%	II
	*CHEK2*	Deletion	Copy number loss	1.4%	II
	*RAD51*	Deletion	Copy number loss	1.2%	II
	*TP53*	Deletion	Copy number loss	1.2%	II
	*TSC2*	Deletion	Copy number loss	1.2%	II
	*CDKN2B*	Deletion	Copy number loss	1.2%	III
	*CREBBP*	Deletion	Copy number loss	1.2%	III
	*CDKN2A*	Deletion	Copy number loss	1.0%	II
	*MAP3K1*	p.R58W	Missense mutation	1.0%	III

ICC, intrahepatic cholangiocarcinoma; HCC, hepatocellular carcinoma.

An in-depth understanding of tumor pathogenesis is crucial for identifying clinicopathological factors associated with tumorigenesis and development. In China, high-risk groups for HCC primarily include those with HBV or HCV infections (19), while ICC is mostly associated with bile duct inflammation, viral hepatitis, intrahepatic bile duct stones, and other diseases (20, 21). Previous reports indicate that chronic liver inflammation is closely associated with multiple primary liver tumors, as most cases involve chronic hepatitis, which plays a key role in primary hepatocarcinogenesis at the molecular level (22, 23). Therefore, any factor causing chronic liver inflammation may be a potential risk factor for sdpHCC-ICC. Studies have shown that sdpHCC-ICC patients in Japan, Europe, and the United States tend to be co-infected with HCV (9, 24), while those in China are more often co-infected with HBV (7). This may be related to geographical differences in virus distribution. In this study, both patients had chronic hepatitis B and were not treated regularly for a long period before the liver tumor was discovered, which facilitated tumor progression. After resection, both patients were on long-term oral antiviral medication, with no recurrence observed to date. This suggests that HBV infection is closely associated with the development of sdpHCC-ICC, and antiviral therapy plays a key role in reducing the risk of tumorigenesis in such patients.

Most sdpHCC-ICC patients present with nonspecific clinical symptoms. In this study, Case 1 was identified as a liver lesion during a routine health checkup. The lesion was initially small, prompting a recommendation for regular follow-up. Subsequent imaging showed lesion enlargement, although the patient remained asymptomatic. In contrast, Case 2 presented with symptoms of abdominal pain and decreased appetite. These findings underscore the nonspecific clinical manifestations of sdpHCC-ICC, which complicate early diagnosis. Routine biochemical tests in sdpHCC-ICC patients are nonspecific. Some patients may exhibit abnormal liver function, such as mild elevation of aminotransferases, correlating with the level of viral load (25). In this study, liver function indices were normal in both patients. sdpHCC-ICC lacks specific serum tumor markers. Since it has components of both HCC and ICC, theoretically, tumor markers AFP and PIVKA II for HCC and CA19–9 and CEA for ICC could be used. Concomitant elevation of AFP and CA19–9 aids in diagnosing sdpHCC-ICC but requires imaging to differentiate it from cHCC-CCA (7, 8). However, the extremely low incidence of sdpHCC-ICC and lack of clinician awareness result in a low rate of preoperative imaging diagnosis. Physicians often diagnose larger tumors as the primary disease and smaller ones as intrahepatic metastases (17). On DCE-CT and DCE-MRI, HCC typically shows “fast-in-fast-out” enhancement (26), while ICC shows peripheral enhancement in the arterial phase, peripheral contouring in the portal vein phase, and delayed enhancement in the central delayed phase (27). The imaging manifestation of sdpHCC-ICC combines both features. Clinicians should consider the possibility of sdpHCC-ICC when observing this pattern, despite its low incidence. Reviewing the imaging data in this study, the preoperative diagnosis in both cases initially considered HCC and overlooked ICC. Therefore, improving understanding of sdpHCC-ICC, considering medical history and tumor marker characteristics, and accumulating experience can enhance the preoperative diagnosis rate.

Surgical resection remains the preferred treatment for sdpHCC-ICC, with the principle of performing radical R0 resection while ensuring residual liver function (8, 28). However, the principle of lymph node dissection differs between HCC and ICC. In HCC, surgery typically requires only liver tumor resection due to the low incidence of lymph node metastasis. In ICC, lymph node metastasis is common, necessitating lymph node dissection (29). Previous studies reported a case where a patient did not undergo lymph node dissection, developed lymph node metastasis after surgery, and eventually died (30). Additionally, extensive use of intraoperative frozen biopsy is recommended for lesions preoperatively and intraoperatively considered atypical HCC with suspected sdpHCC-ICC. HCC and ICC components are located in different tumor foci, independent of each other, with distinct morphologies under the microscope. Hep Par-1 and GPC-3 are reliable markers for HCC, while CK7 and CK19 are valuable for distinguishing ICC from HCC, especially when combined with immunohistochemistry (31, 32). Recently, liver transplantation has been used as a curative option for some patients (33, 34), but its long-term efficacy needs further study. Additionally, ablation therapy is an effective localized treatment for patients with severe cirrhosis who cannot tolerate surgery, offering advantages such as low impact on liver function, minimal trauma, and precise therapeutic efficacy (35). HAIC and TACE are commonly used for unresectable and recurrent tumors, though their use in sdpHCC-ICC is less frequently reported as a complement to surgical treatment (8, 36). Vidili et al. described a case of a patient presenting with jaundice and dyspepsia, diagnosed with sdpHCC-ICC and a concurrent right kidney tumor. They emphasized the critical role of ultrasound technology in tumor diagnosis and minimally invasive treatment, offering valuable insights for managing this rare disease (37). Both patients in this study underwent radical R0 resection followed by postoperative prophylactic TACE combined with chemotherapy or targeted therapy as a comprehensive treatment approach. Case 1 received multiple sessions of HAIC and TACE, given the high risk of tumor recurrence and good treatment compliance, with the aim of improving local tumor control and delaying recurrence. The necessity of postoperative adjuvant therapy, such as chemotherapy, targeted therapy, or immunotherapy, for patients with high-risk recurrence factors still needs further investigation, and this will be the focus of future research.

Few reports exist on the prognosis of sdpHCC-ICC. Available studies suggest that the prognosis is worse than HCC and comparable to ICC (5, 8). Cao et al. retrospectively analyzed the survival prognosis of 35 patients with sdpHCC-ICC and found that the OS at 1, 3, and 5 years after surgery was 60.0%, 28.9%, and 23.1%, respectively. Among these patients, ICC tumor size, lymph node metastasis, and histological differentiation of ICC components were independent risk factors affecting OS (7). Li et al. noted that tumor size affects OS in both ICC and HCC, while tumor size and postoperative prophylactic TACE treatment in ICC also affect DFS (17). We hypothesize that ICC has a greater impact on the survival prognosis of patients with sdpHCC-ICC than HCC. Therefore, more attention needs to be paid to the progression of ICC in clinical practice. The two patients in this study had a good survival prognosis and no recurrence at the time of writing, likely due to their low tumor stage, high degree of differentiation, and prophylactic TACE treatment after surgery. With new advances and breakthroughs in treatment, the survival prognosis of patients has significantly improved. In the future, more multidisciplinary basic and clinical studies are needed to explore safer and more efficient diagnostic and treatment methods. By synergizing diagnosis and treatment and leveraging the professional advantages of various disciplines, we can provide more evidence-based medical evidence and clinical references to improve the prognosis of sdpHCC-ICC patients.

## 5 Conclusion

In this study, we investigated the clinical and pathological features of sdpHCC-ICC through two cases with chronic hepatitis B. The pathogenesis of sdpHCC-ICC is unclear and may involve both monoclonal and polyclonal origins. HBV infection is an important risk factor, and antiviral therapy plays a key role in reducing the risk of tumorigenesis. sdpHCC-ICC lacks specific clinical manifestations and serum tumor markers, resulting in a low preoperative imaging diagnosis rate. Surgical resection remains the treatment of choice, but the prognosis is poor, with the ICC component having a greater impact on prognosis. Postoperative prophylactic TACE, along with adjuvant therapies such as chemotherapy and targeted therapy, plays an important role in improving prognosis.

## Data Availability

The original contributions presented in the study are included in the article/supplementary material. Further inquiries can be directed to the corresponding author.
